# Dynamics Studies of DNA with Non-canonical Structure Using NMR Spectroscopy

**DOI:** 10.3390/ijms21082673

**Published:** 2020-04-11

**Authors:** Kwang-Im Oh, Jinwoo Kim, Chin-Ju Park, Joon-Hwa Lee

**Affiliations:** 1 Department of Chemistry and RINS, Gyeongsang National University, Gyeongnam 52828, Korea; orangekwang@gmail.com; 2 Department of Chemistry, Gwangju Institute of Science and Technology, Gwangju 61005, Korea; wlsdn8810@gist.ac.kr

**Keywords:** nucleic acids, NMR, G-quadruplex, I-motif, triple helix, Z-DNA, dynamics

## Abstract

The non-canonical structures of nucleic acids are essential for their diverse functions during various biological processes. These non-canonical structures can undergo conformational exchange among multiple structural states. Data on their dynamics can illustrate conformational transitions that play important roles in folding, stability, and biological function. Here, we discuss several examples of the non-canonical structures of DNA focusing on their dynamic characterization by NMR spectroscopy: (1) G-quadruplex structures and their complexes with target proteins; (2) i-motif structures and their complexes with proteins; (3) triplex structures; (4) left-handed Z-DNAs and their complexes with various Z-DNA binding proteins. This review provides insight into how the dynamic features of non-canonical DNA structures contribute to essential biological processes.

## 1. Introduction

Nucleic acids play an important role in all biological processes related to genetic information such as replication, transcription, and translation. DNA duplexes typically form a right-handed B-form double-helical structure containing Watson-Crick A⋅T and G⋅C base-pairs. With two complementary strands are paired, RNA preferentially has A-form right-handed double helix. In other cases, RNA can adopt diverse non-canonical structures, such as left-handed helices, i-motifs, triplexes, and quadruplexes as major or minor conformations [1,2,3,4,5]. These structures contain unusual Hoogsteen and/or wobble base-pairs and are key structural components that are essential for the diverse functions of nucleic acids during various biological processes [6,7]. There are comprehensive studies and reviews of the structural studies of non-canonical RNA and base-pairing [8,9,10,11]. 

The non-canonical structures of nucleic acids can have multiple conformational folds, producing a highly dynamic structural ensemble. Even though the less populated conformations are not often visualized in structural studies, they can contribute to folding, stability, and biological function [12,13,14,15]. The exchange process can feature a transient snapshot of biologically important conformational states. Thus, in addition to structural studies of non-canonical nucleic acids, their dynamic features also should be investigated to fully understand the biological function of nucleic acids. Dynamics studies of protein-nucleic acid interactions are complementary to the complex structures, in that they provide detailed information about the binding interfaces, exchange rates, and conformational changes associated with the binding. In this review, we discuss several examples of non-canonically structured DNAs, focusing on their dynamic characterization by NMR spectroscopy: (1) G–quadruplex (G4) structures and their complexes with target proteins; (2) i-motif structures and their complexes with proteins; (3) triplex structures; (4) left-handed Z-DNAs and their complexes with various Z-DNA binding proteins (ZBPs). This review provides insight into how the dynamic features of the non-canonical structures contribute to essential biological processes.

## 2. G-Quadruplex (G4)

**Figure 1:**
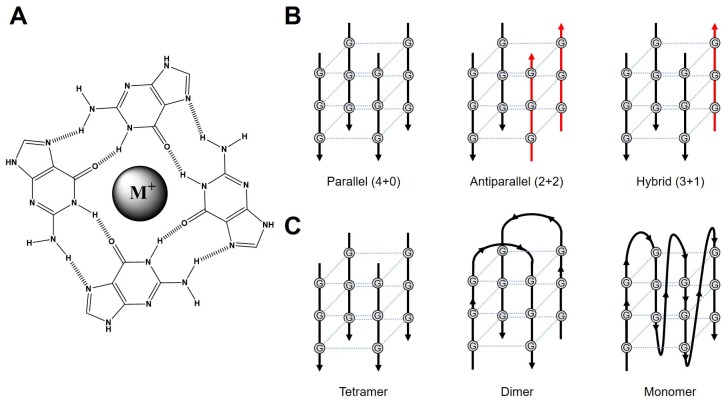
(**A**) G-tetrad formed by four guanines with Hoogsteen base-pairs and a monovalent cation. (**B**) G4 topologies depending on the orientations of the four G-tracts. (**C**) Examples of tetrameric, dimeric, and monomeric G4 structures.

The G-tetrad is a planar structure composed of four guanines with Hoogsteen base-pairs and large monovalent cations in the center of the plane (Figure 1A) [16]. G4s are structures with two or more stacked G-tetrads. Typically, four tracts of three Gs (G_3_) are connected with various looping sequences, while variants with longer lengths of G tracts also form G4s [12,13,17]. Considering its high prevalence in the human genome, especially in oncogene promoters and telomere regions, the understanding of the dynamics of G4s and G4-protein (or ligand) complexes provides valuable information regarding cancers [18,19,20].

G4 structures are conformationally heterogeneous because of the exchange of topology [21], G-register [22], and oligomeric states [23]. Depending on the relative orientations of the four G-tracts, G4 topologies are categorized as follows. The parallel is all four G-tracts are aligned in the same direction (4+0). The antiparallel indicates that two G-tracts are in the same direction, and the other two are in the opposite direction (2+2). The hybrid is that three G-tracts are in the same direction, and one is in the opposite (3+1) (Figure 1B). Loops connecting parallel G-tracts are usually short (1~2 nt) and called propellers. Loops connecting antiparallel tracts are longer (≥3 nt). All four G-tracts can be connected in sequence (intramolecular, monomeric) or can be separate (intermolecular, multimeric (dimeric, or tetrameric)) (Figure 1C). While extensive studies have been performed to reveal the structural aspects of the G4, the contributions of the dynamic properties of G4 are less acknowledged. However, it is becoming clear that the conformationally heterogeneous ensemble of G4s is essential for biological functions such as transcriptional repression in promoters [24].

There are excellent reviews that describe the details of NMR methods for studying the structures [25] and the dynamics of G4s [26]. In this article, we focus on recent dynamics studies of G4s alone and G4s in complex with proteins or other ligands.

### 2.1. G4 DNA

**Figure 2:**
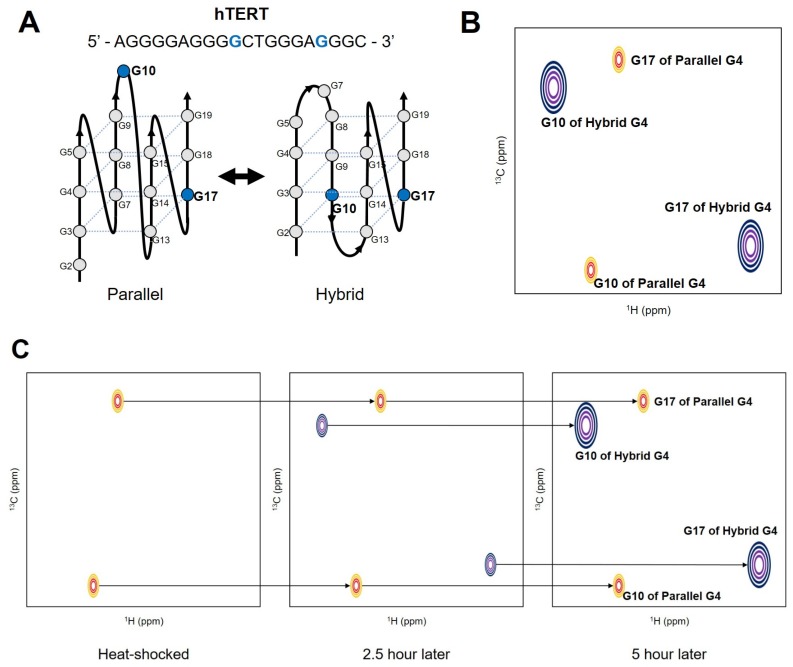
(**A**) G-register isomers of hTERT G4. The ^13^C labeled G10 and G17 are shown in Blue. (**B**) Schematic drawing of ^1^H-^13^C HSQC spectra of hTERT G4 with ^13^C labeled G10 and G17. (**C**) Schematic drawing of NMR monitoring of the re-equilibration process. Redrawn from Ref. [27] by permission of Oxford University Press on behalf of Nucleic Acids Research.

In the G-rich DNA sequence which forms unimolecular G4, four G-tracts are linked with three loops. When more than three G nucleotides are present within each tract, the different numbers of Gs in the four G-strands induce sequence-based dynamic polymorphism [6]. Different conditions of pH, cations, or ligands also can induce polymorphism. Depending on which G participates in the hydrogen-bonding networks of the G-tetrad, different isomers (G-register isomers) can be formed. Figure 2A shows that the G-register exchange can be coupled with topological transitions, as shown in the hTERT promoter sequence.

The base-pair opening occurs during structural fluctuations of DNA. To characterize the dynamic motions of base-pair opening, the imino protons are useful probes in NMR studies [28,29,30,31,32,33]. The exchange of imino protons provides quantitative information on the thermodynamics and kinetics of the open/closed state using a two-state model [30]. A recent study showed that NMR could be successfully applied to reveal the slow exchange process between G-register isomers of c-Myc and hTERT sequences [34]. By using a photolabile protecting group, a single conformation of each G4 was initially trapped, and the restoration of conformational heterogeneity after releasing the protecting group by irradiation was monitored with real-time ^1^H NMR spectroscopy. The time dependence of the signal intensities of the imino protons from each isomer revealed that the re-equilibration occurs on a timescale of hours (the rate constants of exchange were measured as h^−1^). To compare the real-time NMR data with other kinetics experiments, the authors performed thermal hysteresis UV-Vis denaturation experiments to measure the folding/unfolding rate of the representative G4 isomers. K^+^-induced folding measurements by time-resolved NMR spectroscopy were also employed. During isomer exchange, the c-Myc isomers maintain their partially folded intermediate structures, while the hTERT isomers follow an unfolding-refolding mechanism. The re-equilibration of conformational heterogeneity of the hTERT G4 was also monitored with atom-specific ^13^C labeled dGs [27]. By incorporating 8-^13^C-dG at the specific position, the authors observed two ^1^H-^13^C cross-peaks from one guanine, which indicates the existence of two distinct conformations in the slow chemical shift time regime. The re-equilibration process was monitored by measuring peak intensities in Band-selective Excitation Short-Transient (BEST)-TROSY HSQC [35] spectra after heat shock (Figure 2). These studies demonstrated that the inherent conformational heterogeneities of G4s can be successfully investigated with NMR spectroscopy. However, the ^13^C/^15^N labeling techniques and the use of photolabile protecting groups for the DNA sample preparation are not widespread yet. More advances and commercialization of these techniques are required to study the dynamics of G4s with heteronuclear NMR.

Besides the typical G4s with three G-tetrads, G4s formed with four G-tetrads also have conformational heterogeneity. The structural polymorphism of d(G_4_C_2_)_3_G_4_ was recently revealed [36]. The d(G_4_C_2_)_3_G_4_ is the minimal element of the d(G_4_C_2_)_n_(G_2_C_4_)_n_ repeats in the non-coding region of the C9orf72 gene, known for potentially causing the neurodegenerative diseases amyotrophic lateral sclerosis and frontotemporal dementia [37,38]. Two major antiparallel structures were stabilized with 8Br-dG modification at a specific position, and the structures were investigated. Conventional 2D NMR techniques were applied to collect all restraints for the structural calculation. 1D ^1^H NMR spectroscopy was used to identify the population of each form of G4 structure based on the imino and aromatic proton assignments at various temperatures. Interestingly, the population of each conformation was affected by the annealing speed and the pH. Their interconversion was very slow, so the peaks from both conformations were observed in the same spectra. The solution structure contained a pseudo-C-quartet formed by the Cs in the lateral loops on top of the four G-tetrads. This research showed that pH can affect G4 conformation by changing the protonation state of loop residues.

As we showed above, most NMR studies of G4 DNAs routinely monitor imino protons to demonstrate conformational heterogeneity and slow exchange. One noteworthy study used the ^1^H 1D NMR of the imino protons quantitatively. Varizhuk et al. investigated the polymorphism between G4 monomers and polymers by integrating techniques such as atomic force microscopy, optical and electrophoretic analysis, and molecular modeling with NMR spectroscopy [39]. 2D diffusion ordered spectroscopy was used to estimate the molecular weight from the diffusion coefficients, and the data suggested that parallel G4s without overhangs forms oligomers. Also, after integrating the imino proton region of the ^1^H NMR spectra, the total numbers of imino protons were found to be equal to the expected numbers in the oligomeric state. The research we mention here shows that the conformational differences between G4 structures and the exchange between them can be effectively studied with NMR spectroscopy, and we expect that advances in DNA labeling technology, chemical modification, and NMR techniques will contribute to this field. 

### 2.2. G4-Protein Interaction

Many proteins are involved in biological processes such as telomere maintenance, replication, and transcription regulation by interacting with the exceptionally stable G4. The biochemical properties of G4 helicases that unfold G4 structures by ATP hydrolysis and their potential as anticancer targets are well documented in recent reviews [40,41]. Nonenzymatic proteins such as hnRNPA1, hPOT1, and high mobility group B1 (HMGB1) have also been revealed as G4 interactors [42,43,44,45]. NMR spectroscopy has been applied to characterize the binding surfaces, binding affinities, and the exchange timescales between the free and bound forms of the protein-G4 complexes.

**Figure 3:**
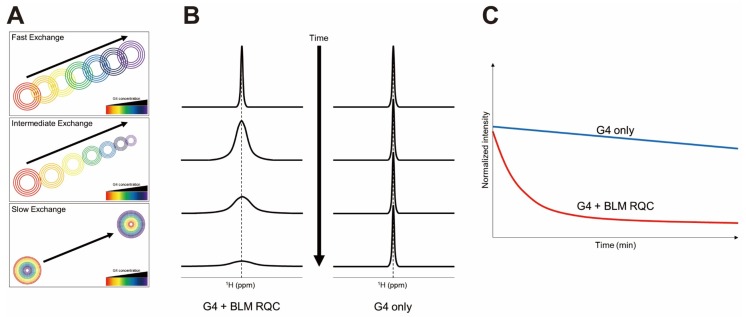
(**A**) CSP patterns with the G4-protein complexes in different exchange regimes on the NMR timescale. Schematic drawings of (**B**) a ^1^H imino proton peak in the middle plane of the G4 in an H/D exchange experiment and (**C**) a decay profile of a specific imino proton.

Amato et al. investigated telomeric G4 recognition by HMGB1, which is involved in telomere maintenance in the nucleus [46]. The peak intensity changes in the ^1^H-^15^N HSQC spectra upon titrating unlabeled DNA into ^15^N-labeled proteins were analyzed. Decreases in peak intensity upon ligand addition are usually considered as evidence of interaction in the intermediate regime of the NMR timescale (Figure 3A). In combination with other biophysical and computational techniques such as surface plasmon resonance and docking simulations, the G4 binding surfaces and binding affinities were revealed. The third RGG motif (RGG3) in the translocated in liposarcoma (TLS)/fused in sarcoma (FUS) protein forms a tertiary complex with telomeric G4 DNA and telomeric repeat-containing RNA (TERRA). Interactions between RGG3 of TLS/FUS and telomeric G4 DNA and TERRA were monitored with NMR spectroscopy [47]. The chemical shift perturbations (CSPs) of the imino protons of the G4 and the amide (^1^H-^15^N) cross-peaks of the proteins were analyzed. Because of the poor spectral dispersion and the overlap of signals from many Arg/Gly residues, only partial assignments of the amide signals from other than Arg/Gly were available. However, the binding surfaces for two G4 nucleic acids are distinguishable based on the different CSP patterns of specific residues. This study showed that the atomic resolution of NMR spectroscopy could provide valuable information about the interaction when the complex structure cannot be obtained.

Intrinsically disordered RGG motifs are found in several proteins. One of the RGG motifs in hnRNPA1 was shown to contribute to specific binding of telomeric G4 [48]. In this study, the CSP *vs* [DNA]/[Protein] ratio was used to determine the dissociation constants, which could not be determined with isothermal titration calorimetry because of the very weak binding affinity. By using various G4 structures as ligands, the authors showed that the RGG motif specifically recognizes the structured loop in the G4. The gradual changes in cross-peaks observed in this study indicate that the RGG motif and its G4 complex are in fast exchange in NMR timescale (Figure 3A). Also, the intensities of the imino protons of the G4 in 1D NMR decreased with increasing concentration of the RGG motif. It was consistent with the results of the G4 unfolding assay performed with circular dichroism (CD) spectroscopy.

The G4 unfolding mediated by helicases contains several steps that accompany structural rearrangements of both G4 and proteins [49,50]. The c-MYC G4 interaction with two human RecQ helicases (Werner syndrome protein (WRN) and Bloom syndrome protein (BLM)) was studied independently [51,52]. The RecQ C-terminal (RQC) domain of WRN was subjected to titration with non-G4 DNA or G4 DNA, and the residues which showed G4-specific responses were identified [51]. Interestingly, many amide peaks in the ^1^H-^15^N HSQC spectra disappeared upon addition of even small amounts of G4 DNA (< 0.05 molar equivalents). In this study, the G4-specific residues were not located in the duplex DNA binding surfaces identified by previous crystal structures [51,53].

In the case of BLM RQC, titration with up to 2 molar equivalents of DNA was performed, and CSPs could be observed, while only a few peaks disappeared upon addition of G4 [52]. Interestingly, the significantly perturbed residues were partially overlapped with the known duplex DNA binding surfaces [52,54]. Further investigation with Car-Purcell-Meiboom-Gill (CPMG) relaxation dispersion experiments showed that the BLM RQC-G4 interactions are in the intermediate regime on the NMR timescale. CPMG relaxation dispersion experiments have used to quantify micro − millisecond time scale dynamics of proteins by analyzing R_2,eff_ on different CPMG frequencies [55]. The exchange rates, populations, and chemical shift differences between different states can be obtained. In this study, H/D exchange experiments were used to monitor G4 unfolding induced by BLM RQC. As expected, the imino protons in the middle plane were observed only after D_2_O exchange, and the decay profiles were obtained per each guanine (Figure 3B,C). The results showed that the D_2_O exchange rate is much faster in the presence of BLM RQC. This study exemplifies a quantitative way to evaluate G4 unfolding by proteins with NMR spectroscopy.

As we described above, most NMR studies of G4-protein interactions have used the amide cross-peaks of the protein and the imino protons of the G4 as the fingerprints. More probes, such as the aromatic ^13^C-^1^H cross-peaks of the G4, could complement the current tools. Also, more detailed dynamics investigations are expected to provide insights into G4 recognition by proteins.

### 2.3. G4-Ligand Interaction

G4 targeted ligands have recently emerged as a promising strategy for developing anticancer drugs. Because telomerase is highly expressed in many kinds of tumor cells, telomeric G4s have been considered as a potential target for ligands that bind to and stabilize the G4 for inhibition of telomerase [56,57]. G4s in oncogene promoters such as c-MYC, c-kit, and KRAS are also important in cancer biology. It is known that c-MYC transcription is upregulated in 80% of solid tumors, and it could be regulated by c-MYC targeted therapeutics [58,59]. There are several recent reviews of the design, synthesis, and therapeutic potential of G4 ligands [18,57,60].

1D ^1^H NMR spectra of G4 imino protons have conventionally been used for monitoring G4-ligand interactions because they detect not only the binding but also more subtle structural conversions. A transition in the folding topology of Tel23 G4 (d[TAG_3_(T_2_AG_3_)_3_]) upon BMVC-8C3O (3,6-bis(1-methyl-4-vinylpyridiumiodide)-9-(1-(1-methyl-piperidiniumiodide)-3,6,9-trioxaundecane) carbazole binding was monitored with time-resolved NMR spectroscopy [61]. Particularly, H/D exchange spectra showed that there was incomplete disruption of hydrogen bonding in the middle plane during the folding transition, indicating that the folding topology conversion is a slow process and not associated with the global unfolding events. Debnath et al. deduced that bis-triazolylcarbazole (BTC) interacts with and stabilizes a minor conformation of the c-MYC G4 by monitoring CSP of imino protons [62]. Together with single-molecule Förster resonance energy transfer (smFRET) data, the NMR data suggested that the G4 stabilization by BTC resulted from conformational selection and not an induced-fit process. The RNA polymerase I inhibitor BMH-21 was tested for c-MYC G4 (Pu22-T14/T23) binding [63]. The imino proton spectra of the G4 with increasing concentrations of the ligand clearly showed that the binding is an intermediate to slow exchange process, and the stoichiometry was also determined as 2:1 (drug: G4). Another anticancer agent, CX5461, was also titrated into c-MYC G4 (Pu22-T14/T23), and a similar change of the imino proton spectra was observed. The pi-pi stacking interaction between the G4 plane and small molecules is known as one of the most crucial binding determinants. However, the staking interaction is not quite specific because the planar base structures are all common regardless of different G4 structures.

There have been efforts to develop ligands that will bind specifically to each G4 structure. Thiazole peptides were developed and tested for specific c-MYC G4 binding [64]. These experiments also showed that the ligand binds to c-MYC’s G4 in slow exchange on the NMR timescale. In this research, imino, anomeric, and aromatic signals of each nucleotide were monitored during the ligand titration, and CSPs were calculated. The data showed that both terminal regions are responsible for ligand binding. The modified peptide (KR-12C) of human cathelicidin formed a complex with c-MYC G4 specifically, and the solution structure of the complex was determined with NMR spectroscopy [65]. The imino proton spectra of the c-MYC G4 with a different version of the peptide (KR-12A) showed that the signals from G4 were broadened and new signals from the hairpin Watson-Crick base-pairs appeared. This indicates that site-specific substitution affects the G4 binding and folding topology conversion of cathelicidin. The research discussed here used various biophysical techniques such as fluorescence spectroscopy, molecular docking simulations, and isothermal titration calorimetry together with NMR spectroscopy. To advance the design of more specific G4 binding ligands, these combined approaches are required. The dynamics of the G4-ligand complexes we have discussed are mostly in the slow regime in the NMR timescale. Combining with the structural heterogeneity of G4s themselves, these features warrant more detailed investigations. 

## 3. I-Motif

**Figure 4:**
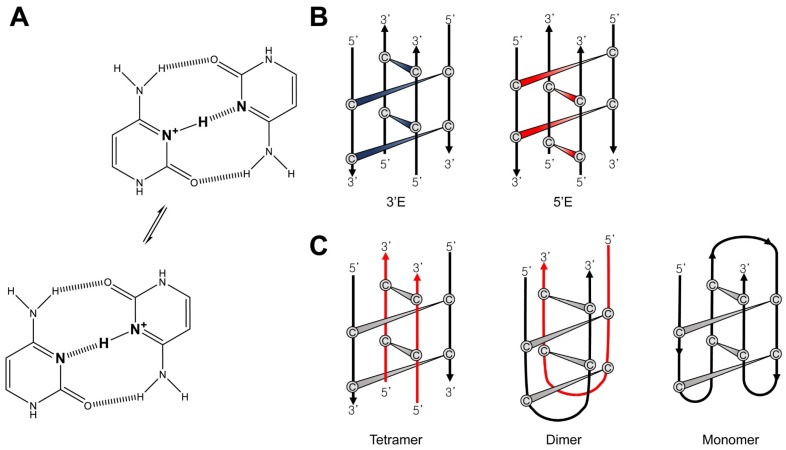
(**A**) Hemiprotonated C⋅C^+^ base-pairs. (**B**) I-motif topologies (5′E and 3′E). (**C**) Oligomeric states of i-motifs.

The i-motif is one of the non-canonical DNA structures that can be built from strands complementary to those that form G4s. It has two parallel duplexes held together by hemiprotonated cytidine and protonated cytidine (C⋅C^+^) base-pairs intercalated in an antiparallel orientation (Figure 4A) [66]. Because of the requirement of protonated bases, the formation of i-motifs is known to be more stable at acidic pH conditions [67]. The length of i-motifs is dependent on the number of C⋅C^+^ base-pairs [68]. As there are large numbers of G4s in human promoters and telomeres [69], it is expected that i-motifs are also abundant in cells and that they, like G4s, are involved in the regulation of gene expression [70,71]. Previously, the biological relevance of the i-motif was suspected because of its poor stability at physiological pH and a lack of direct evidence that i-motifs actually existed in the intracellular environment. However, a recent study revealed the presence of an i-motif in vivo using NMR techniques [72]. Dzatko et al. found that the C-rich DNA sequence forms an i-motif in human HeLa cells, and the i-motif has better thermostability in vivo compared to in vitro, based on in-cell 1D ^1^H NMR imino spectral data. Although there is a difference in the origin of the base-pair compared to G4s, the i-motif also has conformational heterogeneity depending on its topology [73] and oligomeric state [74]. Based on the position of the outermost C⋅C^+^ base-pair, i-motif topologies can be classified into two types. When the C⋅C^+^ base-pair is nearer to the 5 ′end of the DNA strand, it is called a 5’E topology. Conversely, if it is closer to the 3 ′end, it is a 3′E topology (Figure 4B) [75]. The number of DNA strands that make up the i-motif determines its oligomer state. I-motifs can be generated from one strand (intramolecular, monomer) or from more than one strand (multimer, dimeric or tetrameric) (Figure 4C).

There are reviews of the structural studies of the i-motif [75] and i-motif binding ligands [76]. As a complement to these, we summarize the NMR studies of the structural and dynamic properties of i-motifs.

### 3.1. I-Motif DNA

To form i-motifs, a C-rich DNA sequence must have multiple C⋅C^+^ base-pairs. The C⋅C^+^ base-pair is formed by hydrogen bonding in which the nitrogens in the 3-position of the two cytidines have a hemiprotonated N∙∙∙H∙∙∙N moiety [66]. Previously, it had been proposed that N∙∙∙H∙∙∙N bonding of the C⋅C^+^ base-pair would follow the hypothesis of either 1) a symmetric single-well potential or 2) a delocalized proton oscillating between two wells with a double-well potential [77]. Lieblein et al. demonstrated which of the two hypotheses is correct through NMR spectroscopy [78]. The ^1^H-^15^N HMQC spectra showed that the ^1^H chemical shifts of the two nitrogens forming the C⋅C^+^ base-pair were identical, meaning that they share one proton. Also, it was found that the proton rapidly moves between two nitrogens through observation of the ^1^J (NH) coupling constant. Thus, the C⋅C^+^ base-pair, which is the basis of the i-motif, follows the pattern of hydrogen bonding characterized by a proton hopping between two nitrogens like hypothesis 2) (Figure 4A).

The slow association rate and strong binding capacity of dimeric i-motif d(5mC_2_TCACTC_2_)_2_ were characterized using NMR experiments [79]. Restraints for the structural calculation were obtained through conventional 2D NMR methods. At a slightly acidic pH (pH 6.8), d(5mC_2_TCACTC_2_)_2_ had a dimeric topology. This was confirmed by the similarity of the imino proton spectra with that of the known d(5mC_2_TCICTC_2_)_2_ [80]. Monitoring base-pair lifetimes through imino proton exchange experiments revealed that C⋅C^+^ base-pairs contribute to the structural stability of the i-motif. In particular, it was found that the C⋅C^+^ base-pair at the core of the i-motif has an extremely long lifetime. It is similar to the dimer topology lifetime, meaning that the innermost C⋅C^+^ base-pair plays an important role in the maintenance of dimerization. In addition, analysis of dimer-monomer exchange kinetics revealed that the dimer stability has a pH dependency and the i-motif has an association rate four orders of magnitude slower than the formation rate of B-DNA duplexes.

Esmaili et al. investigated tetrameric structures of two versions of *Tetrahymena* telomeric DNA repeats, d(A_2_C_4_), and d(C_4_A_2_), by 2D NMR methods [81]. While the d(A_2_C_4_) tetramer had a single 5’E topology, it was found that the d(C_4_A_2_) tetramer shared two topologies. A series of TOCSY experiments was conducted to determine the ratio of the two topologies. It was found, by monitoring the intensities of the H6-H5 cross-peaks of the spectra, that the ratio of the 3’E topology increased compared to the 5’E topology as the temperature increased. However, the ratio of the two topologies did not depend on pH conditions or oligonucleotide concentrations. According to the base-pair opening kinetics analysis using proton exchange, the lifetime of the innermost C⋅C^+^ base-pairs was significantly longer than that of the outer base-pairs. 1D NMR imino proton observation was conducted to identify whether the i-motif lost its tertiary structure due to the formation of G-C base-pairs when presented with G4 in solution. After d(A_2_C_4_)_4_/d(C_4_A_2_)_4_ + d(T_2_G_4_)_4_ was stored for one month, broadening of the imino proton peaks of the i-motif and G4 did not occur. Rather, broadening of the adenine proton peaks and fast exchange rates of thymidine imino protons were observed, indicating a temporary interaction of terminal adenosines and thymidines.

The application of NMR to the vertebrate telomere sequence, d(C_3_TA_2_)_3_C_3_, enabled the study of conformational heterogeneities of the i-motif with pH [82]. d(C_3_TA_2_)_3_C_3_ was identified as having both 5’E and 3’E topologies through ^1^H-^15^N HMQC experiments. Using time-resolved NMR spectroscopy, i-motif folding and kinetic partitioning were observed with decreasing pH in proportion to time. Through monitoring the imino proton region by 1D NMR, it was found that a change in topology from 3’E to 5’E could occur depending on pH. In addition, fast exchange rates were shown for folding into both topologies, but the exchange between the two topologies was very slow in the NMR time scale. This experiment showed that pH can influence the topology of the i-motif.

As mentioned above, heterogeneous structures and dynamics of the i-motif were routinely studied by monitoring chemical shift changes of imino protons under various conditions. It is expected that more non-canonical DNA structures and their properties will be revealed using NMR spectroscopy.

### 3.2. I-Motif Binding Ligands and Proteins

**Figure 5:**
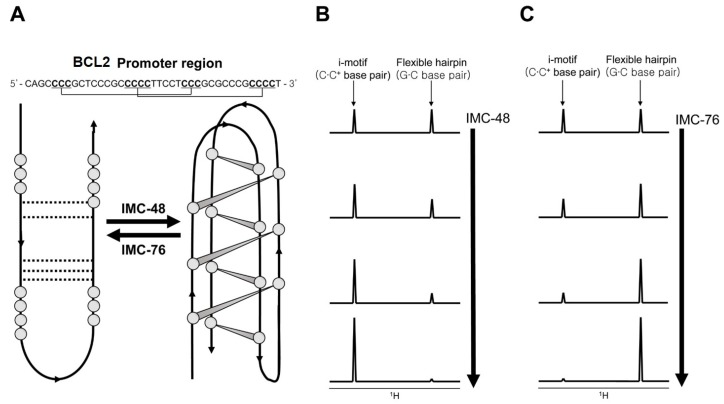
(**A**) The structural conversion from monomeric to dimeric BCL2 C-rich strand induced by ligands. Cytidines participating in C⋅C^+^ base-pairs for i-motif formation are marked in bold. (**B,C**) Schematic drawing of ^1^H imino proton peak of BCL2 C-rich strand changed by ligand titration.

The discovery and design of i-motif binding ligands is not well developed due to the fact that its formation occurs in acidic pH in vitro [76]. Starting with TMPyP4 [83] and BisA [84], a few ligands that can bind to i-motifs have been described [76,85]. Among them, a study on ligands for the C-rich DNA sequence of the human B-cell lymphoma gene-2 (BCL2) promoter region is relatively well described [86]. Kendrick et al. discovered BCL2 C-rich sequence-specific binding ligands IMC-48 and IMC-76, which respectively induce formation of the i-motif and flexible hairpins, by FRET high-throughput screening assays (Figure 5A). After that, it was revealed by imino proton peak monitoring through 1D NMR that the topology heterogeneity of the BCL2 C-rich sequence changes with the addition of ligand. When IMC-48 was added, the imino proton peak corresponding to the i-motif was prominent (Figure 5B), but in the case of IMC-76 addition, the peaks of the flexible hairpin were observed (Figure 5C). Even in the state where one ligand is bound to DNA, a change of topology occurred by increasing the concentration of the other ligand. Actually, the shifting of the equilibrium in one direction by the ligands occurred in vivo, during their regulation of the expression of the *BCL2* gene.

The search for i-motif binding proteins has been very limited. In vitro biochemical assays revealed that the hnRNP K protein and the ASF/SF2 splicing factor bind to the telomeric C-rich strand, but no further structure-based studies have been conducted [87]. Currently, hnRNP LL is the only protein whose binding region for the i-motif has been identified [88]. The binding of the RRM1 and RRM2 domains (RRM12) to the P1 promoter of BCL2 contains a 39-mer C-rich sequence (Py39wt) that was evaluated using NMR experiments. ^1^H-^15^N HSQC spectra showed that the i-motif binding site of RRM12 is retained at physiological pH (pH 5.5–7.5). Interestingly, this binding was in the intermediate regime on the NMR timescale at low pH but changed to a slow regime when the pH increased. Further ^31^P NMR and ^1^H NMR experiments revealed that the conversion of the Py39wt i-motif to the duplex form due to the rise in pH causes a change in the RRM12 chemical exchange.

Structural and kinetic studies of i-motif binding ligands/proteins have been stagnant compared to studies on G4s. However, as it was revealed that the i-motif exists in vivo [54], it is expected that the research on the i-motif and its binders using NMR spectroscopy will be actively conducted in the future.

## 4. DNA Triplex

**Figure 6:**
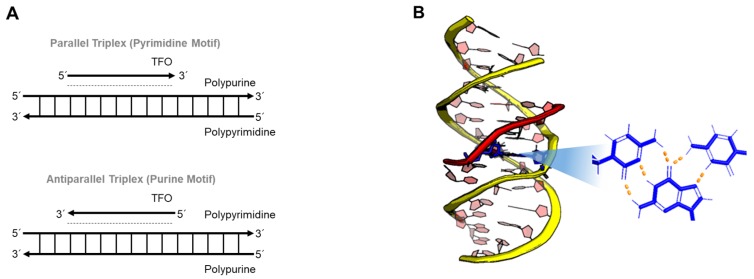
(**A**) Illustrations of parallel and antiparallel triple helices (TFO, triplex-forming oligonucleotide). (**B**) Side-view of a triple helix and base interactions at its midpoint (PDB id = 1BWG). The backbone of the TFO is shown in red. H-bonds between base-pairs are indicated by orange dashed lines.

Major groove recognition of a homopurine and homopyrimidine DNA duplex by a third strand results in DNA triple helices (triplexes). Since the first discovery of a triplex in 1957, Dervan and coworkers showed the broad potential of triplex-forming oligonucleotides (TFOs) to inhibit sequence-specific DNA-protein interactions [89,90,91]. As described in Figure 6, the composition of the TFO, which uses Hoogsteen base-pairing, influences the orientation of a triplex. In the case of pyrimidine-rich (or purine-rich) oligonucleotides, the triplex forms a parallel structure (or antiparallel structure), so-called YRY (or RRY). Triplex DNA can be formed not only by the binding of a third strand to a DNA duplex, it can also be induced by homopurine-homopyrimidine mirror repeats pulled out of the upstream regions, so-called H-DNA [92,93]. The name of H-DNA originated from the fact that triplexes preferentially form under acidic conditions [6]. 

Triplex structures have been an area of interest in biological research for several decades because of their biological roles in processes such as mutagenesis, lytic replication of the Epstein-Barr virus genome, and homologous recombination [94,95,96]. They also have potential applications in biotechnology and molecular medicine. To understand the biological roles of triplex DNA and the applications of TFOs, it is essential to explore triplex structure and dynamics, so that the molecular interactions between bases in solution are well defined. 

The stability of DNA triplexes is strongly influenced by base sequence, length of the strands, and strand mismatches, as well as solution conditions, such as temperature, salt concentration, and pH [97,98,99,100]. How the third strand associates with or dissociates from a duplex has been studied using varying approaches, such as fluorescence spectroscopy and simulations [99,100,101,102,103,104]. For instance, base-stacking interactions in TAT triplex and AT duplex DNA were compared by fluorescence decay, and showed restricted motional dynamics in the TAT triplex compared to the AT duplex [105].

The exchange rate of protons using 1D NMR can be a useful technique to elucidate site-specific kinetics and dynamics of DNA triplexes. Russu and coworkers investigated the base-pair opening dynamics in DNA triplexes using imino proton exchange rates, and found that the stability of Hoogsteen base-pairs in a DNA triplex is comparable to that of Watson-Crick base-pairs in a DNA duplex [106]. They also reported that a GTA triad has destabilizing effects on its neighboring triad, though it has similar stability to a TAT triad [107]. Later, the exchange rates of individual Watson-Crick and Hoogsteen imino protons were reported by the monitoring of water magnetization transfer and hydrogen/deuterium (H/D) exchange in the presence and absence of Mg^2+^ ions [108]. The H/D exchange measurement provided kinetic information on the slower structural opening reactions than kinetics determined by water magnetization transfer experiments. The results showed the energetic effects of ions at individual sites of DNA. They suggested that Mg^2+^ ions stabilize the closed conformations of base-pairs since Mg^2+^ ions are closely associated with the DNA. Recently, selective binding between triplex DNA and intercalators has been investigated, and the triplex DNA was shown to undergo covalent attachment based on CSPs in ^1^H-^15^N HSQC spectra and NOE data [109,110]. These studies elucidated sequence- and pH-dependent stability, showing a more stable TAT triplet at the 5’ junction under low pH, and slow exchange of chemical shifts between the coexisting species.

## 5. Z-DNA 

One of the notable features of DNA is its ability to transition between right-handed B-DNA and left-handed Z-DNA, which has a zigzag backbone with syn- and anti- conformations [111,112,113]. The B–Z transition of DNA is known to happen in a sequence specific manner, occurring in regions enriched with purine-pyrimidine repeats, (GC)_n_ [112]. It has been found that Z-DNA is present in vivo and plays important roles in biological functions, such as remodeling of chromatin structure, activation of transcription, and binding of proteins during viral infection [114,115,116,117,118]. To understand the kinetics and dynamics of Z-DNA, it has been extensively investigated using a broad range of methods, such as single-molecule techniques and NMR spectroscopy [119,120,121,122,123], while steady-state methods, such as X-ray crystallography, CD measurements, fluorescence spectroscopy, and 1D NMR results, have been used to determine the structural features of the B–Z transition [111,124,125,126,127]. In particular, hydrogen exchange measurements by NMR spectroscopy, which measure base-pair opening kinetics, have emerged as a promising technique to characterize the fluctuations of the inter- and intramolecular interactions of the bases and Z-DNA binding proteins (ZBPs) [33].

### 5.1. Z-DNA

Among the various non-canonical DNAs, Z-DNA has received attention due to its exceptional left-handed helical conformation, which shows a more elongated helical structure than B-DNA and different chirality [111,112,113]. The repeated units of (CG)_n_ or (GT)_n_ are the most common sequences forming Z-DNA, with the free energy cost for switching from B-form to Z-form ranging from 0.33 to 0.66 kcal/mol/bp [128,129]. The structural propensity to form Z-DNA within repeated sequences is as follows: d(CG) > d(TG/AC) > d(GGGG) > d(TATA). However, the formation of Z-DNA is also possible in sequences that lack pyrimidine-purine repeats or that contain AT base-pairs [130,131,132,133]. Based on numerous experimental and theoretical studies, it is widely known that the B–Z transition occurs under conditions of high ionic strength, negative supercoiling, and protein binding [134,135,136,137]. Tashiro et al. found that the Z-DNA conformation was favored at low temperature [138,139]. In addition, methylation of DNA, such as 5mCpG or 8mCpG methylation, greatly favors transitions from B-form to Z-form at physiological salt concentrations [120,140,141,142].

Based on the proton exchange data, the rate of base-pair opening of a Z-DNA duplex consisting of CG sequences was reported to be approximately five times smaller than B-DNA composed of same sequences, while B-DNA base-pairs are known to open on 1–10 microsecond timescales [31,143,144].

**Figure 7:**
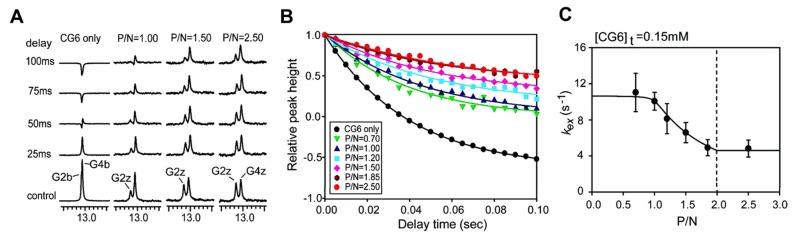
(**A**) One-dimensional imino proton spectra of the water magnetization transfer experiments. P, protein; N, nucleotide. (**B**) Relative peak intensities in the water magnetization transfer spectra for G2z imino resonances as a function of delay time. (**C**) Exchange rate constants of the G2z imino protons as a function of the hZα_ADAR1_:d(CG)_3_ molar ratio (P/N ratio). Reprinted with permission from Ref. [145]. Copyright 2009 American Chemical Society.

Figure 7A shows the 1D NMR imino spectra from a water magnetization transfer experiment for a 6 base-paire (6-bp) DNA duplex, d(CG)_3_ (also called CG_6_), with/without ZBPs, as described by Kang et al. [145]. The exchange rate k_ex_ for the CG_6_ duplex is determined using the relative height of the imino resonances as a function of the delay time after water inversion. Figure 7B shows gradual changes of the G2z resonance of d(CG)_3_ and d(CG)_3_-protein complexes. The relative peak intensities are fit to equation 1, which extracts k_ex_ values based on the best fit (Figure 7C).

(1) $$\frac{I\left(t\right)}{I_{0}}=1-2\frac{k_{ex}}{\left(R_{1w}-R_{1a}\right)}\left(e^{-R_{1a}t}-e^{-R_{1w}t}\right)$$

The exchange rates of the G2b and G4b imino protons of the d(CG)_3_ duplex were determined as 21 and 12 s^-1^, respectively. As the ratio of protein increased in the DNA-protein complexes, the imino protons of the Z-form are exchanged more slowly, indicating an increase in stability of the duplex. Dynamics and kinetic models of DNA-protein complexes will be discussed in the next section.

### 5.2. Z-DNA Complexes with ZBPs

ZBPs and antibodies can selectively bind to Z-DNA (or Z-RNA), inducing conformational transitions. Crucial biological functions of Z-DNA are induced by association with ZBPs, examples being the RNA editing enzyme ADAR1 [137,146,147], the DNA-dependent activator of IFN-regulatory factor (DAI, also known as DLM-1 or ZBP1) [148,149], the poxviral E3L protein [118,150], and RNA-dependent protein kinase (PKZ) [151]. These ZBP and DNA complexes adopt well-defined structures that have been elucidated by crystallographic and NMR studies [152]. Interestingly, despite the wide variety of biological functions, there are structural analogies between ZBPs. In each case, the residues in the α3 helix and β-hairpin (β2-loop-β3) of ZBPs mediated the intermolecular interactions for DNA recognition. Recent studies of the structures and biological functions of Z-DNA-ZBP complexes provide insights into DNA recognition and B–Z transition induced by ZBPs.

Recent NMR studies suggested an active mechanism of B–Z transition, which support two independent roles of ZBPs as follows [123,145,153,154,155]: (i) one ZBP molecule binds to 6-bp B-DNA, d(CG)_3_, and facilitates the conversion from B-DNA to Z-DNA; (ii) the second ZBP forms the complete DNA–(ZBP)_2_ complex. The Zα domain of human ADAR1 (hZα_ADAR1_), which stabilizes the Z-DNA conformation, is able to bind to a 6-bp Z-DNA duplex with not only CG-repeat-rich sequences but also non-CG-repeat sequences [156]. The results of hydrogen exchange experiments of imino protons for free d(CG)_3_ and hZα_ADAR1_–d(CG)_3_ complexes elucidated the overall slower exchange rates of imino proton in the Z-form versus the B-form [145]. A kinetic model demonstrated that active-mono B–Z conversion bridged the equilibrium between B-form complex (BP) and Z-form complex (ZP). Recently, Lee et al. carried out the structural dynamics analysis of hZα_ADAR1_–d(CG)_3_ complexes based on the global fitting methods of relaxation dispersion combined with CSP [155]. ^15^N CPMG relaxation dispersion results showed that hZα_ADAR1_ with Z-DNA undergoes a pseudo-three-state conformational exchange, which includes two independent B–Z transitions for the free and bound states. The Zα domain of yatapoxvirus E3L (yabZα_E3L_) exhibits efficient B–Z conformational changes of d(CG)_3_ like the hZα_ADAR1_–d(CG)_3_ complex, while the Zβ domain of human DAI (hZβ_DAI_) –d(CG)_3_ complex undergoes lower-efficiency B–Z transitions [157].

**Figure 8:**
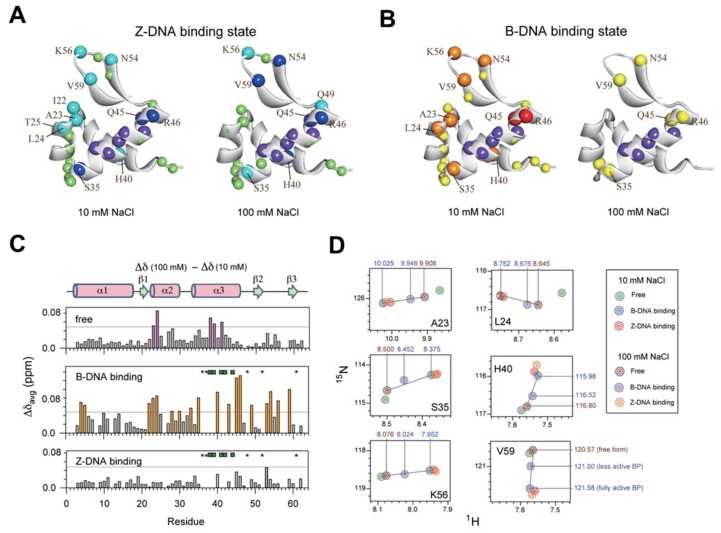
(**A,B**) DNA binding patterns of (A) Z-DNA and (B) B-DNA binding at 10 (left) or 100 mM NaCl (right). Chemical shifts are illustrated by different colors: red or blue, >0.18 ppm; orange or cyan, 0.12–0.18 ppm; and yellow or pale green, 0.08–0.12 ppm.The purple spheres indicate sites with disappeared or very weak cross-peaks during titration. (**C**) The average chemical shift differences between [NaCl] of 10 and 100 mM with free caZα_PKZ_ (upper) and caZα_PKZ_ – B-DNA (middle) and Z-DNA (lower) complex (**D**) The calculated ^1^H-^15^N HSQC cross-peaks of caZα_PKZ_ – B-DNA or Z-DNA complexes in 10 or 100 mM NaCl solutions.

Using the same methods as above, including water magnetization transfer, CSPs in ^1^H-^15^N HSQC spectra, and CPMG relaxation dispersion experiments, the B–Z transition mechanism and quantitative energy landscape of the Zα domain of PKZ from *Carassius auratus* (caZα_PKZ_) have been investigated [154]. The caZα_PKZ_ is able to transform d(CG)_3_ to a Z-DNA conformation with lower activity rates than hZα_ADAR1_ and yabZα_E3L_. The caZα_PKZ_–DNA complex, which has a Z-DNA conformation, induced significant CSPs for the α3, β1- β2, and β-hairpin regions at different salt concentrations, due to the reduced B-Z transition activity at high salt (see Figure 8). Taken together, despite the structural similarity of varying types of ZBPs, the complexes between different ZBPs and DNA have different kinetic and dynamic effects on B-Z transitions of DNA duplexes.

During B–Z transition, the most notable change is the shift in chirality between a right-handed and left-handed structure. Thus, as a common method, monitoring of the B–Z transition has been determined by inversion of the signs of CD spectra [124,158,159,160]. Recently, using a time course of signals recorded at 255 nm, the B–Z transition rate of the caZα_PKZ_–DNA complex has been reported [161]. In this study, more significant changes of the time traces for caZα_PKZ_–DNA than hZα_ADAR1_–DNA suggested the crucial role of charge-charge interactions in the B-Z transition. Since fluorescence is strongly sensitive to the solvation environment, structural torsions, and the distance between fluorescence acceptor/donor pairs, fluorescence spectroscopy is one of the most useful methods for monitoring the change of DNA and DNA complexes [120,121,123,124,125]. Lee et al. investigated supercoiling-induced B–Z transitions at the single-molecule level using smFRET combined with magnetic tweezers, and suggested that torsion plays an important role in the B-Z transition [122]. We anticipate that these spectroscopic methods will be a compatible partner with NMR to understand the biological functions induced by the dynamics of ZBP–Z-DNA complexes.

## 6. Conclusions

The non-canonical structures of DNA are essential for their diverse functions during various biological processes. These non-canonical structures can undergo conformational exchange among multiple structural states. Their dynamics data illustrates that each conformational state can play important roles in folding, stability, and biological function. Currently, NMR can be one of the most versatile tools for studying DNA dynamics and DNA-ligand interactions at the atomic level. The NMR techniques that allow monitoring of different time scale motions have been frequently employed. This review provides insight into how the dynamic features of the non-canonical structures revealed by NMR spectroscopy comprehensively can be understood. Further dynamics studies are expected to give a more detailed view of the dynamic landscape of the non-canonical DNA.

## Abbreviations

G4	G-quadruplex
BEST	Band-selective Excitation Short-Transient
HMGB1	High Mobility Group B1
RGG3	The third RGG motif
TLS	Translocated in liposarcoma
FUS	Fused in sarcoma
TERRA	Telomeric repeat-containing RNA
CSP	The chemical shift perturbation
CD	Circular dichroism
WRN	Werner syndrome protein
BLM	Bloom syndrome protein
RQC	RecQ C-terminal
CPMG	Car-Purcell-Meiboom-Gill
BMVC-8C3O	3,6-bis(1-methyl-4-vinylpyridiumiodide)-9-(1-(1-methyl-piperidiniumiodide)-3,6,9-trioxaundecane
BTC	Bis-triazolylcarbazole
smFRET	single-molecule Förster resonance energy transfer
C∙C^+^	Cytidine and protonated cytidine base-pair
BCL2	Human B-cell lymphoma gene-2
RRM12	RRM1 and RRM2 domains
TFO	Triplex-forming oligonucleotide
ZBPs	Z-DNA binding proteins
DAI	DNA-dependent activator of IFN-regulatory factor
PKZ	ZBP-containing protein kinase
hZα_ADAR1_	Zα domain of human ADAR1
yabZα_E3L_	Zα domain of yatapoxvirus E3L
caZα_PKZ_	Zα domain of PKZ from *Carassius auratus*
